# Co-creating innovation for sustainability

**DOI:** 10.1007/s11612-022-00619-8

**Published:** 2022-01-24

**Authors:** Jörn Erbguth, Marianne Schörling, Nathalie Birt, Susann Bongers, Pamina Sulzberger, Jean-Henry Morin

**Affiliations:** 1 Institute of Information Service Science—CUI, Geneva School of Social Sciences, University of Geneva, Geneva, Switzerland; 2 Geneva Macro Labs, Geneva, Switzerland; 3 Geneva, Switzerland; 4 bcompanion gmbh, Lucerne, Switzerland

**Keywords:** Innovation, Design, Sustainability, Impact investing, Design thinking, Agile development, Collective intelligence, Systems theory, Systems thinking, Sustainable innovation, Innovation, Design, Nachhaltigkeit, Impact Investment, Design Thinking, Agiler Entwicklungsprozess, Kollektive Intelligenz, Systemtheorie, Systemisches Denken, Nachhaltige Innovation

## Abstract

This contribution to the journal Gruppe. Interaktion. Organisation. (GIO) presents a case study for an approach to design sustainable innovation. No nation is on track to achieve the UN sustainable development goals for 2030. The traditional innovation ecosystem is insufficient. Rather than only solving problems, technological innovation is creating new challenges that society is struggling with. Innovation needs to be developed differently to focus on impact.

Geneva Macro Labs initiated a new approach to foster sustainable innovation which was based on a combination of systems theory, collective intelligence, agile development and design thinking. The initiative, called *Geneva impACTs,* brought together a diverse group of experts, start-ups and investors to develop innovative projects, aiming to make inroads towards achieving the Sustainable Development Goals 2030. It started at a time when COVID-19 measures made it impossible for groups to meet in person and so the entire process was conducted virtually using a range of online tools.

A critical reflection shows the methodological strengths of the Geneva impACTs approach and identifies suggestions for improvement to be considered for future iterations. As an overall result, this new methodology is highly conducive to impact innovation.

## Abstract (de)

Dieser Beitrag der Zeitschrift Gruppe. Interaktion. Organisation. (GIO) stellt anhand einer Fallstudie einen Ansatz zum Design nachhaltiger Innovation vor. Die Ziele der Vereinten Nationen für nachhaltige Entwicklung bis 2030 zu erreichen, scheint keiner Nation zu gelingen. Die bestehenden Innovationsmechanismen haben sich als unzureichend herausgestellt. Statt bloß Probleme zu lösen, schafft Innovation zusätzliche Herausforderungen, mit denen die Gesellschaft zu kämpfen hat. Daher muss Innovation darauf fokussiert werden, die Nachhaltigkeitsziele zu fördern.

Geneva Macro Labs initiierte und verfolgte einen neuen Ansatz zur Förderung nachhaltiger Innovation, der auf einer Kombination aus Systems Theory, kollektiver Intelligenz, agiler Entwicklung und Design Thinking basiert. Die Initiative *Geneva impACTs* brachte eine Vielzahl von Experten, Start-ups und Investoren zusammen, um innovative Projekte zu entwickeln, die einen Beitrag zum Erreichen der Ziele für nachhaltige Entwicklung 2030 leisten sollen. Als die Initiative ins Leben gerufen wurde, machten COVID-19-Maßnahmen es den Beteiligten unmöglich, sich persönlich zu treffen und zwang sie dazu, komplett via Online-Tools zu interagieren.

Eine kritische Reflexion zeigt die methodischen Stärken dieses Ansatzes und identifiziert Verbesserungsvorschläge, die bei künftiger Anwendung berücksichtigt werden sollten. Im Ergebnis können Innovationen, die Nachhaltigkeit zum Ziel haben, sehr von dieser neuen Methodik profitieren.

## Introduction

Innovation holds great promise to make the world a better place. In recent times, the lives of many people have been transformed by innovation, though often the driving force for innovation has been financial gain to the detriment of larger, important societal considerations.

Due to the rapid expansion of the world’s population, globalization, and industrialization, humankind now faces a set of new and acute challenges that require urgent and innovative action. These challenges are both complex and significant: many transcend national boundaries, affect multiple sectors of society and impact billions of people. If we are to successfully address these issues, given their scale and complexity, new approaches to innovation must be developed to achieve tangible, timely and sustainable solutions. Innovation for sustainability could help the world’s population raise global living standards, improve human health and protect the environment (Soumitra et al. 2021).

Against this backdrop, innovation for sustainability has been gaining importance, not only as a narrative to guide the changes towards new socio-technical and socio-ecological systems (Patterson et al. 2017), but also as a tool to strengthen preparedness, coordinate responses across multiple sectors and mitigate critical risks.

Since the 1990s, the integration of sustainability principles into business has been on the rise on the corporate agenda (Hoffman 2018, Bonini and Görner 2011). In 1997, Elkington coined the notion of sustainable development in terms of the *Triple Bottom Line*, in which the private sector is urged to adopt a responsible approach and incorporate environmental and social dimensions in addition to economic dimensions in decision-making. Elkington (1998), and Adams et al. (2016) showed how companies could introduce ‘eco-efficient’ optimization of organizational processes as sustainability-oriented innovation. Henderson (2020) makes the case that “sustainability is an innovation problem” referring to the urgency of “reimagining capitalism due to a world on fire”, whereas Henderson (2006) highlights that sustainability can be driven when conventional economic models are replaced with approaches where multi-disciplinary policies and appropriate metrics are utilized.

Despite the increasing integration of sustainability principles in business and its rise in importance on the corporate agenda (Hoffman 2018, Bonini and Görner 2011), sustainable innovation processes and outcomes are often left wanting for lack of cross-sector collaboration with open innovation. Even in Switzerland, which was recognized as the world’s most-innovative economy in 2021 in the Global Innovation Index (Soumitra et al. 2021), *sustainability* and *responsible impact* tend not to be key considerations to fund start-ups—new business ventures or new commercial or industrial projects—which can be effective drivers of (open) innovation processes. Understandably, being highly dependent on external investment, start-ups tend to focus primarily on return on investment. The size and newness of start-ups can be seen as a double-edged sword; being small and free from long-established systems and processes, start-ups can be agile and more easily adapt to challenges through innovation (Bogers 2011), however, their relatively limited resources can leave them vulnerable and many fail. The current innovation ecosystem also gives rise to the probability that start-ups may be developing ideas in isolation, potentially unaware of synergies with other stakeholders and unable to benefit from the wisdom of collective intelligence.

So begs the question: How can sustainable innovation be galvanized and supported to successfully meet the challenges of today? Under which conditions are innovative initiatives likely to thrive and result in the development of market-fit solutions to address sustainability challenges?

In response to this pressing question, and to bring about tangible, timely and sustainable development, the Geneva Macro Labs, which is a think-and-do tank, created a start-up initiative called *Geneva impACTs. *The hypothesis was that start-ups, coupled with the collective intelligence of a think tank community, could co-create sustainable innovation by building on and combining each other’s strengths. Through Geneva impACTs a new business model founded on co-creation for the initiation and development of sustainable solutions was crafted and tested.

This paper outlines the benefits for combining several approaches and principles to boost innovation towards sustainability and presents systems theory as theoretical point of reference. First, the approaches used for the different innovation phases are discussed. *Collective intelligence* served as a foundation for the innovation process, which started with a *Design thinking* workshop. *Agile development* and *systems thinking* were principles applied to adapt to a dynamic and evolving ecosystem. The authors also consider the compatibility of innovation processes in a richly diverse multi-stakeholder setting. Existing studies indicate that forming a relationship with partners is paramount to start-up success (Teece 2010). In the Geneva impACTs case study, potential investors, subject matter experts (as external advisors), and innovators were brought together to develop solution concepts. Based on the principle of systems thinking, the authors reflect on how the development of impact innovation can be best supported and they highlight the benefits and some challenges that may come with collective intelligence.

## Problem description

As an international body that is meant to be the foremost forum to address issues which transcend national boundaries, the United Nations promotes a considerable number of innovative programs and products. The 2030 Global Development Agenda directs its work and calls the world to “leave no-one behind” by implementing 17 Sustainable Development Goals (SDGs) with their 169 targets (United Nations General Assembly 2015). The agenda provides a framework around the pressing issues of our times that require immediate action and changes from all aspects of society, the public, private and civic sector. The SDGs imply different concepts of transitions and transformations, which suggest that technological innovations paired with relevant narratives can provide a set of distinctive sustainability-oriented solutions, which can shape pathways of systemic change. However, despite the efforts made, as the situation stands, no nation is on course to achieve the SDGs by 2030 (Sachs et al. 2021) and the COVID-19 pandemic has contributed to another setback for sustainable development (ibid.). Ideas for sustainability need to be implemented to create impact and successful innovation requires connecting ideas with teams and investors. One key research question remains: How can innovative action be supported best to accelerate the *2030 SDG Agenda*?

Collaboration across all sectors is a way to operationalize the universal nature of the global sustainability agenda. Co-creating innovation that focuses on sustainability aims to address sustainability issues; bringing different stakeholders together has reached almost a paradigmatic status in the public and private sector (van Hille et al. 2020). However, fragmentation of responses, ineffective partnerships (Pattberg and Widerberg 2016), and poor ownership to measure SDG indicators, inter alia, challenge the international community’s ability to take effective action (Gennari and Kalamvrezos Navarro 2020).

Based on the presented explorative case study, the authors argue that the co-creation of innovation for sustainability requires specific structures to thrive, such as clear frameworks for collaboration and instruments to connect the “right” set of stakeholders, establish trust between them and help them reach agreement on their sustainability objectives. These elements are open innovation measures (Chesbrough 2003) which are often studied from a macro level and rarely from an operational level (Bigault de Casanove 2020, p. 14).

What approach best supports a diverse community engaging in an open innovation process towards sustainability, where problems can be connected with solutions at all stages? How can the tension between collaborative knowledge sharing and competitive protectionism of ideas and insights be managed so as to avoid risking potential innovation falling by the wayside during the process or being blocked by expertise (Bogers 2011)? To learn from and build on the first iteration of the Geneva impA*C*Ts initiative, a qualitative study based on evaluative interviews with independent facilitators who were each responsible for supporting and overseeing one of three different, interdisciplinary innovation focus groups was conducted.

## Components of a methodology to create innovation for sustainability

Given the complexity of addressing sustainability issues and technological innovation simultaneously, methods which focus on just one dimension seem insufficient. For this reason, Geneva Macro Labs united a community of professionals from many different sectors, all with the shared belief in the importance of the long-term goals of sustainable development. They applied a systems-oriented approach, which incorporated the knowledge and perspectives of a wide range of stakeholders, in order to gain a better understanding of how different components of an innovation methodology could be combined.

### Systems theory

Systems theory provides the theoretical foundation for designing an innovation methodology as it takes into account the very characteristics of open innovation communities (Tani et al. 2018). These characteristics include the interdependence of its members, the presence of a feedback system to regulate them as systems, and the necessity to adapt to external stimuli.

As an analytical tool in the realm of innovation, system theory refers to the distinction between a system and its environment. There are many diverse branches of systems theory, which historically cover the three areas of mechanical, biological, and social. Within these three areas, different and often conflicting schools of thought have been developed (Saake and Nassehi 2007, Carayannis et al. 2016). The common central thread in systems theory is that a system consists of individual elements, their interdependence and interaction.

In a sociological context, communities may be described as “systems” and the interpersonal behaviour or “functions” of each individual within them can be seen as co-forming the structure. By changing elements of the system, for example, the communication between the individuals, the system can be reshaped. By contrast, the branch of structural functionalism, which explores how system structures determine the behaviour of individuals in a society, argues that functions of a society are extremely stable and can only be changed by external factors. Such definitions can help us to conceptualize how innovation (systems) can respond to today’s challenges, as innovation can be seen as a “knowledge-concept” (Carayannis et al. 2016, p. 9) that brings the political, economic, education, and the research and development (R&D) systems together.

### Systems thinking

The application of systems theory, otherwise known as *systems thinking*, helps to handle complex situations and problems by identifying patterns and rules within them (Mulgan 2021). As a project management principle, systems thinking encourages innovation project managers to define early on, what levels of control and formalization of communication the project needs in order to succeed (Kapsali 2011).

Systems thinking can assist to take a big picture view of complex environments, for example, innovation ecosystems or large organizations. In other words, systems thinking can help innovators appreciate operational flexibility of their ecosystem by identifying and using boundary management activities to understand and potentially re-shape their system; on a practical level this could help them adjust to the economic factors of their environment and find ways overcome difficulties of limited resources.

Systems thinking can be seen as referring to people’s ability and willingness to think beyond the borders of an existing system, recognize the interrelatedness between individuals within the system, and start to ‘play’ with the borders and rules of several systems. This is possible when experts with different specializations and backgrounds come together and create a common room of systems thinking. Collaboration of this nature can spark spontaneous and creative idea generation that can energize the participants and both broaden and enrich the output of the innovative process. Where potential gaps in perspectives arise, facilitators who apply systemic methods help bridge them by asking hypothetical questions (Daimler, Sparrer, and Varga von Kibéd 2016).

From the outset, systems thinking underpinned the Geneva impACTs initiative; instead of aiming to control the outcome of the innovation cycle, the team focused their efforts on adapting to the behavior and needs of the innovation teams themselves and turned to methods known from social innovation and software engineering.

### Agile development

Evidence suggests that conventional management practices, such as detailed planning, formalized communication and restrictive managerial action to handle change are not conducive to the success of innovation projects (Kapsali 2011). In other words, innovation processes which include measures for adjusting to changing demands of their environment are more likely to contribute to the success of a project. In light of these findings, Geneva impACTs used elements from agile development to support their open innovation process.

The evolution of software development might be able to convey important learnings for society. Technically speaking, software engineering is completed once the software requirements have been defined in a formal and complete way and can be interpreted by a computer. While architects need construction workers to build what they have designed, software is automatically executing plans. The relative simplicity of software engineering compared, for example, with building engineering allows software to tackle more complex problems. While developing highly complex software, software engineers realized that project management needed to be revolutionized to reduce the risk of failure for software development projects.

Software development has long followed the *waterfall model* (Balaji 2012), which is rigidly structured in sequential phases; the next phase is only commenced once the prior phase has been finished. As such, the detailed design is completed before any lines of code are written. *Obergfell* at the SCRUM-Institute claims that the waterfall model is to blame for many software project failures since in large software projects the majority of requirements tend to change (Obergfell 2011). Although in other areas society is fighting failures by increasing documentation requirements, control and strict compliance to the project plan, this does not work for software development. Software development did not follow this line and was revolutionized when the *Agile Manifesto* was born (Beck et al. 2001).

The Agile Manifesto is based on the idea of facilitating change rather than trying to prevent deviation from a plan (Fowler and Highsmith 2001); the ability to react to unpredictable events is considered more important than trying to plan for disasters. Getting early feedback is as important as constantly adjusting priorities. While in the waterfall model, originally planned functionality is prioritized over change requests, agile development constantly adjusts the plan according to new priorities. The development of a software project is a learning exercise; during the course of the project, knowledge about the requirements and the knowledge about the ability of software to model them continue to increase. Early prototypes, minimal viable products and close contact to the customer support that learning curve. Discovering design flaws early reduces lost effort compared to fixing flaws later. Things that can be decided later, should be decided later, because deciding later means deciding while being wiser. *Timeboxing* which gives priority to available resources rather than to a predefined plan enables resources to be used more efficiently.

Agile project management is increasingly used in contexts outside software development. It has been proven to be superior to traditional project management when projects involve many unknowns. In other situations, a hybrid approach might be favorable (Ciric et al. 2018). Increased regulation can be a challenge to the use of agile development methods (Mehrfard and Hamou-Lhadj 2011). The EU is trying to foster and shape digital innovation in its Digital Agenda with a flood of regulation. It started with the General Data Protection regulation, GDPR (Regulation (EU) 2016/679) and is now being followed by the Artificial Intelligence Act, Digital Markets Act, Data Act and the Digital Services Act. Crypto assets will be regulated by the coming MICA regulation and the revised eIDAS regulation will serve as a legal basis for digital identities (European Commission. Directorate General for Communications Networks, Content and Technology 2020). Regulatory compliance imposes processes that often conflict with agile innovation. Although this regulatory approach is intended to increase trust, ethics, and sustainability of innovation, it runs the risk of becoming a barrier to innovation in general, including innovation for sustainability.

### Design thinking

Innovation is dependent on finding creative solutions. Design thinking is one way to define problems and to find solutions in an iterative and human-centered way. It focuses on trying to better understand the end user, challenging assumptions, and redefining problems so as to find creative new solutions that might not be immediately apparent to our initial level of understanding (Johansson-Sköldberg et al., 2013; Brown and Katz 2009; Liedtka 2015).

The development and testing of multiple and rapid iterations during the process is a way to clarify and define the real problem and empathize with the end user’s needs and experience of the output. This process is particularly useful in addressing complex problems that might be ill-defined or misunderstood. The main goal is to identify and define real problems and any underlying issues in order to create real solutions and real innovations, as opposed to creating products and services that nobody will use or needs.

There are five phases in design thinking, which—in practice—do not need to follow any particular order. They do not need to run sequentially and can even occur simultaneously and repeat iteratively.

- ***Empathize*** with the people the product or service is being designed for. What are their pain points and challenges? What do they care about? There are several methods to deepen understanding of the end user, such as conducting interviews and using personas, which represent a specific group of people with particular characteristics.
- ***Define*** the problem and the user’s needs. This is done by analyzing the information obtained in the first phase (or other phases of the process) in terms of what the people described and getting to the root of their real needs from their perspective, instead of basing the problem definition on our own assumptions. At the end of the second step, a problem statement can be formulated.
- ***Ideate*** through brainstorming exercises to come up with many ideas that could potentially solve the problem formulated in phase two. In this phase, it can be very useful to already gather feedback from the end users on some ideas, for example by sketching and showing them.
- ***Prototype*** something simple that can be tested. The focus is set on a particular idea from step three; however, it is not yet the final product but a very basic version of it that allows for change.
- ***Test*** the prototype with real people and get feedback in real-time while avoiding explaining how it works or defending the idea. The feedback should be as authentic as possible in order to understand what works and what needs improving. From there, go back to step three and four and apply the feedback and learnings. This process is repeated until the prototype solves the real problem.

In practice, this process is usually applied in “a system of spaces” rather than in this predefined order (Brown 2008). It means that the search for a solution is motivated by the process and particularly by looking beyond the surface and finding inspiration from the users who encounter the problems instead of our own mindset.

### Collective intelligence

*Co-creating innovation for sustainability* requires incremental strategies that facilitate coordination and policy influencing and allow for shared sustainability objectives. Hence, in order to cater to societal needs while acknowledging boundaries of the world’s ecosystem, innovation trajectories and sustainability pathways need to be aligned. This approach accentuates collective intelligence as a prerequisite for innovation that supports sustainable development.

*Collective intelligence* is frequently used as synonym for *swarm intelligence, wisdom of crowds*, and *crowd science*, as well as with methods such as open innovation and crowdsourcing (Noveck 2021, p. 173). While the Greek philosopher Aristoteles already looked at achieving better results through engaging more people in decision-making in Athenian polis as a “middle way” between independent-guess aggregation and deliberation (Ober 2013), today’s scholars look at collective intelligence as a means of dealing with uncertainty and creating the “best” solution for challenging problems. Collective intelligence unites people with different backgrounds and terminology. This diverse crowd could turn into a tower of Babel. Collective intelligence involves the challenge to turn this Babel tower into a lighthouse.

Research of (Hong and Page 2004) suggests that under specific circumstances, a random group of intelligent problem solvers will outperform a group of the best problem solvers (see ibid, p. 16389). In other words, in a problem-solving context, diversity within a group prevents its members from becoming too similar and positively affects their ability to perform well. Diversity helps to counter the trade-offs of group thinking, including how easy possible counterarguments are addressed (Surowiecki 2004). Other conditions that seem to support good group intelligence are independence and private judgement. Recent evidence also suggests that “combining independent decisions substantially increased performance relative to average individual performance” (Kämmer et al. 2017). In addition, under certain conditions, negotiated group judgments can even outperform averaged individual judgments (Bonner and Baumann 2012). Different techniques such as encouraging critical thinking (Postmes, Spears, and Cihangir 2001) or allowing for smooth communication may improve collective intelligence further. Woolley and Aggarwal (2020) documented that “groups that communicated more were more collectively intelligent, but groups in which one or two people dominated the discussion and activity were less collectively intelligent” (ibid., p. 5). Such insights help to develop collective intelligence driven do tanks where subject matter expertise meets entrepreneurial spirit and sustainable finance—three ingredients that can be effectively combined by open innovation.

## Case study—concept & organization

### Project description

Think tanks generally aspire to provide an alternative to the current practice of using people’s intellectual power to formulate new ideas. Instead of replicating closed innovation processes, think tanks respond to particular circumstances by: a) reflecting on how decisions are taken and policies adopted, b) questioning mainstream thinking, and c) enabling disruptive solutions built on innovation and relevance. The latter represents a learning that system thinking and agile development requires a closed loop between ideation, prototypes and implementation. Geneva Macro Labs therefore, positioned themselves not only as a think-tank but also as a do-tank to develop a new methodology of co-creating innovation for sustainability. The study on the Geneva impACTs initiative focuses on the analysis of its methodology and links it to the components mentioned above. The narrative nature of presenting this case study is the result of our qualitative evaluation through interviews with independent facilitators and external experts. Their feedback on the progression of the project and its challenges was solicited during two review phases.

*Impact projects *are initiatives which have the goal of developing sustainability-oriented innovation (see Ginzo 2021). They address the question of how to operationalize global needs and ambitions into marketable solutions (see Schörling and Günther 2019). Their goal is to demonstrate ways to expand sustainable development beyond the traditional social, environmental and governance aspects (ESG) by building and acting upon a *Doughnut Economics* (Raworth 2012) based business model through using the SDGs as a reference.

With the SDGs in mind, the Geneva Macro Labs started the initiative Geneva impACTs. Over a period of twelve months, the group tested a new innovation for sustainability approach in Switzerland with the support of a development fund, the *Migros Pioneer Fund *who acted as an innovation partner. The above methodologies and methods have been selected as tools to generate innovation for sustainability. Together with experts and investors, Geneva impACTs developed specific project concepts that contribute directly to the UN 2030 Agenda and ensure that they have the financing they need. The process comprises two phases: The first is an ideation and build phase, where actual project ideas emerge from design thinking sessions inspired by the UN Sustainable Development Goals (SDGs); executable concepts are built from market-fit ideas by teams of experts working in focus groups that could benefit from a think tank community. The second phase is about venture building and scouting the right execution team for the concept. As such, the “Geneva impACTs process” followed a new investment and venture builder approach, coined as *Impact Creating Investment*.

### Project organization

“People first” meant finding many experts from many different backgrounds and Geneva proved to be a very fertile ground for this. Experienced professionals working in international organizations, non-governmental organizations, corporations and scientists were invited to participate in the Geneva impACTs innovation process. All shared the common goal to foster sustainable development through innovation and engaged as volunteers. The majority of experts had already participated in sustainability focused events. Conferences on different topics like *blockchain for impact* at the UN Palais de Nations and a series of after-work events and webinars brought them together. As a result, when they were individually invited to join the first round of this endeavor, 53 experts signed up through an online form.

The first round started with a design thinking workshop, co-facilitated by experts from academia and the Geneva Macro Labs. Using an online meeting scheduling tool, the best time for the workshop could be identified while providing the organizers with a first overview of the group’s diversity. Despite the challenge of limited time availability of most experts, 38 experts participated in the online creativity activity. The workshop structured interaction between experts by providing different formats: plenary online sessions, so-called *breakout rooms* for several groups, as well as an instant messaging chat room for the facilitators. An online notice board which was able to feature images, links, documents, notes, all collated on a “wall” which was used to take notes.

Six ideas and themes for innovation emerged from the design thinking workshop and the experts voted on which three ideas should be developed in the next phase of the process. Three focus groups were set up to work on the chosen ideas and the experts decided which one they each wanted to be a part of. The task of each focus group was to research and shape the idea into a business proposal that creates impact and is economically self-sustainable. Eventually, the groups worked either on valuing natural capital through tokenization, a new concept on transparency within the packaging industry with a focus on recycling, or a local economy approach for agricultural products. The project team identified and established partnerships to ease the projects’ implementation. Every focus group had a public landing page and a private page with links to the resources. An online drive repository for the collaboration on documents and a web whiteboard with zoomable canvas and sketching functionality were supplied. Each focus group elected their chairperson and co-chairperson.

Each focus group benefitted from the services of an independent professional mediator (and a backup if needed) to facilitate their work. Focus groups met at least once a week via an online conference meeting platform, but often also met more frequently. Where they felt they lacked expertise relevant to their project, focus groups invited external experts to share information and insights. Some people left the focus groups and others joined at a later stage. In all groups, the chairperson, co-chairperson and facilitators ensured continuity throughout, and all focus groups presented well-elaborated concepts. The groups were given three months to complete the draft report on their innovation concept. During the last of these three months, they exchanged draft concepts with investors and the jury to obtain feedback to optimize their concept.

The final reports were then presented to a wider group of 20 jurors, who submitted their questions through an online form. This was provided to the focus groups, to allow them to prepare their answers in anticipation of the final jury session, which was conducted by video conference with breakout rooms to allow for private jury discussions and deliberations.

Investors were invited to all stages of the process. This was important to help develop ideas into feasible and relevant solutions. Potenzial investors could be seen as “customers” of the ideation; being one of the key stakeholders in successful impact innovation, investor needs and concerns need to be addressed to get their buy-in.

## Implementation

### Geneva impACTs innovation process

Innovation processes usually start with ideas and ideas need creativity to come up. Creativity on demand can, however, be difficult. *Online* creativity on demand is even more challenging. Creativity often needs time, to stimulate many senses, human dialogue and inspiration. The Geneva impACTs team considered how they could create an innovation process that would allow for connecting seeds of ideas with concrete solutions, connecting experts from all sectors within an online environment.

**Fig. 1:**
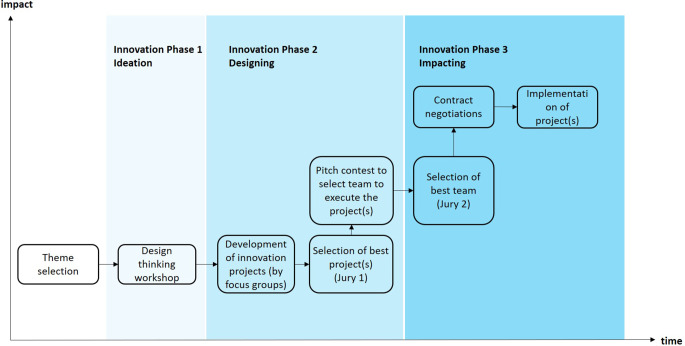
The innovation process applied by Geneva impACTs

The innovation process was organized in three phases, starting with the ideation (phase 1) which was followed by the designing phase (phase 2) and finally the impacting phase (phase 3) (Fig. 1).

For the first phase of the innovation process an innovation infrastructure was built in an iterative manner and allowed for process adaptation as required: (a) the overall innovation theme was selected to frame the initial design thinking exercise, (b) experts were invited to elaborate together ideas and received at the beginning of the design thinking process a short briefing on current challenges within the proposed theme, (c) ad-hoc groups were formed to identify areas for innovation opportunities by allowing for building on individual ideation as a group, (d) voting exercises lead to a list of potential topics for interdisciplinary focus groups, (e) groups pitched their ideas which were subsequently voted on. This rapid design thinking process ended with six project ideas.

The second phase started with selecting three of these project ideas. For these project ideas focus groups were created with a clear mandate to develop a sustainability-oriented innovation, governed by an expert agreement and managed by group-chairs. Within the selected overall theme of “environment and nature at risk”, one out of the projects focused on broadening the perspective on the economic value of ecosystems, notably Great Whales among many others in the oceans and on the shores that are a key factor for oceans to capture carbon emissions. This project was particularly challenging as the scope of the project was difficult to anticipate and many unknowns around the innovative idea of Blue Carbon Credits arose. Learning from case studies on open innovation in SMEs presented in literature (Bertello et al. 2021), several measures were planned for: To avoid the hurdle of inadequate information sharing, the focus groups were supported with communication tools and professional mediators facilitated their online meetings; to address the challenge of time pressure, deadlines were slightly extended; to meet a potential lack of goal redefinition, a template for conceptualizing the project was provided.

Throughout this second innovation process phase, the organizers presented the draft concepts of the focus groups to potential investors, start-ups, and organizations in the public sector to create visibility and identify potential synergies. Crowdsourcing was utilized to attract the engagement of an international community and to raise awareness of this co-creating innovation process within civil society, the public sector and among potential investors. In a jury process all three focus group concepts were evaluated on their relevance, sustainability implications and technological and financial viability, the implementation parameters for the projects were prepared. Investors tended to be happy to give valuable advice in the jury process, though were more reluctant to participate in the focus groups themselves. Participating only in the jury process implied less time commitment for investors but made it more complicated to reflect their ideas and concerns in the process. The second phase completed with scouting the right execution team for one of the concepts that was developed. Those who develop a great idea—particularly using collective intelligence—may not be willing to dedicate themselves, and also, may not be the best to execute the idea. Teams from the start-up scene were invited to provide their proposal for implementing the presented project. At the same time, the finances for implementation were defined and secured through an investor agreement document.

The selection of teams and their proposals by a jury started the third innovation phase. They were partnered with the investor and their ecosystem to implement the project. The implementation is still ongoing. Geneva Macro Labs will continue to support the project through impact controlling to ensure that the focus on the creation of impact is not lost.

### Facilitation of focus groups

After the design thinking workshop and formation of the focus groups, a first meeting, without facilitators, took place online in which the chairperson and co-chairperson in each group were elected. Given the challenges of effective communication between people who have little or no prior experience of working with each other, and recognizing the importance of all group members feeling able to contribute to the discussion in an online forum, Geneva impACTs invited professional mediators to facilitate the meetings and discussions of the focus groups. As the first focus group meeting had been recorded, the mediators were able to gain some understanding of the group dynamics before their first involvement as facilitators. Each focus group was assigned two facilitators, who met their respective co-chairs in a brief introductory virtual meeting to clarify each other’s expectations and their vision for how the group would collaborate effectively.

For some groups, the diversity of the group members and the invitation of different external experts to some sessions needed a lot of flexibility and openness. The level of leadership of the chairperson impacted the dynamics within the group, as did the definition and interaction of the roles of the chairperson and the facilitator. During the co-creation process, facilitators focused on helping their assigned focus group to identify what was needed and when to move the process forward within relatively tight timeframes. Within that context, the following aspects were especially relevant:

- ***Clarifying roles and expectations****:* In addition to the group member roles such as timekeeping or notetaking, it was important for the chairperson and facilitator to discuss and agree who would lead the online meetings. This led to defining mutual expectations and understanding whether the chairs expected or wanted the facilitators to ask questions, coach them, lead the weekly meetings. In each focus group, the facilitator’s role was different and adapted to the group and the chairs’ needs and requirements.
- ***Structuring the discussion****:* To make the most of the time that was available, facilitators and chairs prepared the agenda and structure for each focus group meeting. Meetings tended to last between an hour and an hour-and-a-half and during this time the facilitators endeavored to enable each group member to share their ideas, questions and suggestions while maintaining the forward direction of the discussion.
- ***Maintaining communication and momentum outside the focus group meetings****:* Facilitators maintained regular contact with their respective chairs in between the focus group meetings to discuss progress, ideas for moving forward, addressing any concerns raised between group members and making sure the project was on track. The facilitators also had a weekly facilitators’ feedback meeting with the Geneva impACTs team during which they shared experiences and ideas for improvement for the next iteration of the initiative.
- ***Involvement of the project team****:* Having the support of the Geneva impACTs team at every stage of the process was key to manage dynamics within the focus groups: In addition to providing much needed context, focus, clarification and momentum to the focus groups and their facilitators, they also enabled valuable information to flow between the investors and the focus groups.

The facilitation of the expert focus groups in some ways resembled a jam session, which is not unusual for innovation (Bjelland and Wood 2008). High level musicians improvising together—one has to have a coordinating role; everyone brings their best commitment and experience. Everyone needs to listen to one another and give little non-verbal signals due to the online context. The result was much more than the addition of each part. The role of the facilitator in this context is dynamic and highly dependent on what is happening. Are there any potential misunderstandings or conflicts starting to develop? Is the discussion constructive or is an intervention needed to redress the situation? What would be a good next step? What needs clarification?

### Impacts of Covid-19 measures

The Geneva impACTs initiative and the implementation of the focus groups started during the second wave of the COVID-19 pandemic. First experiences of online workshops and seminars during the first lockdown showed that co-creation and even mediation are possible online with the right tools at hand. At the same time, it also showed how challenging these sessions can be compared to conducting them in person. The Geneva impACTs focus groups embodied this challenge: How much am I seen and heard as an expert? Is my knowledge integrated in the whole process? Do I feel like I am adding value? How much do I put my ego forward?

Working effectively together in an online setting, within the context of a complex environment with multifunctional and diverse teams is based on the assumption that everyone is participating with good intentions and a collaborative attitude. Throughout the process, participants adapted to the online co-creation environment, despite some people being more digitally savvy than others. The support system by the project team allowed for supporting the teams in the uptake of the different collaborative online tools that were proposed. The focus groups used the opportunity to record group meetings for those members who missed them and shared next steps in the team chat.

Innovation is an intense human interaction endeavour. Brainstorming is an integral part of it. In order to visualize ideas, the groups used a virtual whiteboard that was provided by Geneva impACTs. Only a fraction of the team members struggled with new software features; challenges were quickly addressed through one dedicated tech expert. Meeting fatigue was fought against by the establishment of regular jour fixes with clear agendas.

## Evaluation—lessons learned

### Innovation process

Each of the underpinning methodologies played an important and complimentary role during the innovation process and in its successful completion.

Systems theory was instrumental in providing an overarching framework in which methods for enabling innovation were applied. Applying systems theory as part of an initial analysis of the complex innovation ecosystem helped identify patterns of behavior, communication, and interdependence between stakeholders. Seeking to reshape the innovation ecosystem, Geneva impACTs brought together multiple stakeholders and diverse individuals to generate new understanding of complex problems and create new ideas on how to tackle them.

In future cycles of the innovation process, systems thinking could further be used to help identify patterns of fragmented efforts in the sustainable innovation ecosystem and to try to unite them to strengthen their impact and increase their progress towards sustainable development goals.

In hindsight, difficulties sometimes arose when team members representing different systems with different contexts collided: Different work styles let to different ways and expectations how to deal with disagreements in a constructive manner accepted by members of the group. Chairs and co-chairs had a very high level commitment while several other participants showed varying levels of dedication for this unpaid work. Sometimes rules and behaviors that were consistent with one system, did not fit as well within another. This led to a degree of misunderstanding, which slowed progress in the initiative and may have impacted on the level of confidence that individuals within one system had towards the other. One such example was the communication from the focus groups towards the jury of impact investors. In this situation the “learner” or “innovator” needed to understand the context of the situation and the appropriate communication and action. The two different groups or systems did not easily manage to create a new and common pattern. One reason could have been that the jurors entered the innovation process at a later stage.

The analysis of the Geneva impACTs initiative suggests that implementation of collaboration guided by SDGs benefits from involving numerous stakeholders—public and private sector entities, and civil society at all stages of innovation processes. Diverse groups of people were brought together to support the emergence and implementation of innovative ideas through connecting idea generators, impact investors and those with expertise to implement. Such an approach can be summarized as a collective intelligence driven one and requires a large group of experts. Initially, the process started with 53 experts, which proved to be an appropriate size, but a larger group could be envisaged in future cycles and additional stakeholder groups could be brought in. Depending on the nature of the innovation concept, visiting different locations and diving into different communities in a series of combined events with academic institutions, civil society, government agencies and industry could be an effective way to deepen understanding of the problem being addressed and generate ideas for sustainable solutions.

The impact investors played an active and valuable role in the latter stages of the innovation process. Their early feedback and questions on the initial innovation project drafts helped the expert focus groups to start developing an understanding of the impact investors with their key interests and concerns, which informed their subsequent efforts. In future cycles, it could be helpful if impact investors were to join the innovation process at an earlier stage as this may better bridge the gap between different systems and help focus groups develop concepts that meet the needs and interests of all key stakeholder groups. Investors could even fully integrate into the focus groups. From the systemic point of view, the patterns and rules of the “investors’ system” are different from that of the experts. To create and bring together both patterns for innovation, better integration and communication of “new roles” would be beneficial and consideration should be given to the framing of roles, which are likely to impact on the group dynamics.

When planning the innovation process, there was the hypothesis that co-creating innovation would be key to developing tangible impact but was little knowledge of how this novel approach would actually evolve. Against a backdrop of uncertainty, agile development was used to manage the dynamic innovation process, which enabled the project milestones to be met and successfully completed, despite unexpected challenges, including the outbreak of COVID-19, which meant the plans had to be adapted, some even before the process had started.

A conscious decision was made to set tight milestones and final project deadline. The rationale being that more experts would be able to actively participate if their involvement was over a couple of months instead of a longer period. Furthermore, having more time does not mean people will work or produce more, there is rather more time for procrastination. “Time boxing” is a concept that is known to work in agile development, and it was effective in this first cycle to maintain momentum and the focus of those involved. In future cycles, more iterations and additional tools like hackathons could be added, to allow revisiting ideas from different angles, building on them and fostering creativity. Intervals should remain short between milestones as with longer timeframes you risk losing people along the way. During the early stages of the innovation process, design thinking was successfully used for ideation, although the process would have benefited from more time for iterations of the other design thinking phases, such as prototyping and testing. This was made even more acute as the session was conducted online rather than in person. In future cycles, allowing more than four hours for the design thinking process would enable a deeper focus on the problem and the needs early on. This should help the expert focus groups define the problem and scope of their innovation more efficiently and effectively. Prototyping and testing their innovation concepts would also likely improve the investment readiness of their ideas.

Collective intelligence was an important factor of success in the Geneva impACTs initiative. The diversity of the experts’ background, specialization and experience enabled different perspectives to be shared and explored, leading to the development well-considered concepts. Sometimes during the process, other experts from Geneva Macro Labs’ think-and-do tank network who had relevant and complementary experience were invited as guests to share insights which were highly beneficial to the focus groups. Alone, no single expert would have achieved what the focus groups managed to achieve through collective intelligence.

Consistent with the findings of Woolley and Aggarwal (2020, p. 5) focus groups in which there was a high level of interaction and where the discussion was balanced rather than being dominated by one or two members, demonstrated higher collective intelligence. Inviting highly specialized experts as guests, when needed, enhanced the discussions. In fact, the focus group with the most active and communicative members developed the innovation concept that received the best evaluation by the jury.

### Focus group facilitation

Using professional mediators to facilitate the focus groups proved effective in creating a constructive forum for experts, many of whom had never met, to collaborate and co-create. Despite the tight project deadlines and the fact that all meetings and interaction were conducted online, each focus group, with the support of the facilitators successfully developed and completed their concept presentation on time.

The facilitators improved the communication and understanding between individual group members and provided the opportunity for different perspectives to be heard and discussed. As independent neutrals, the facilitators were able to help the focus groups manage conflicting views and maintain momentum during the sessions and during the entire process when the experts were doing deep dives into their fields of expertise. The facilitators were also instrumental in providing continuity when chairs or co-chairs were unable to attend due to unforeseen circumstances.

Although the facilitators were able to watch a recording of the first focus group meeting before they conducted their first facilitation, it would have been helpful if they had been introduced and involved from the outset. Setting the stage by explaining everyone’s roles and agreeing how the group will work together would help manage expectations and avoid potential confusion, frustration, lost time and disharmony. Likewise, discussing and understanding roles, objectives and expectations at an early stage is essential between facilitators and the Geneva impACTs team to ensure success. In future iterations, consideration could be given to having a companion throughout the entire process who could scout, observe and better facilitate the interactions of patterns, rules and contexts from a macro perspective rather than from the circle of the focus groups.

While the online facilitation of the focus groups was effective and made it easier for people to meet, the level of commitment to the project by individual focus group members may be increased if the introductory meeting was held in person so that they start to build connections, understanding and rapport. Another possibility could be to conduct a hybrid format (a combination of virtual and physical) to have everyone who plays an important role on board.

### Context of pandemic—working online

Working online became the new normal due to COVID-19 lockdowns and restrictions and this significantly shaped the way the project was carried out. The upsides of the project being entirely online were that travel times were eliminated, it was easier to arrange meeting times, and experts from all over the world who would otherwise have been inaccessible were willing and able to participate. The value of the knowledge sharing and insights provided by experts in an online setting was sufficient and possibly just as effective as if it was done in person. However, communication, interaction and collaboration within focus groups and processes of co-creation were more challenging online; the level of understanding was reduced as people’s ability to read the group was limited by their screen. In addition, people experienced fatigue after shorter periods of time when participating in focus group discussions and co-creation. These experiences highlight the question of which aspects of the process are well suited to an online setting, which possibly less so, and how to approach the next iteration of the initiative.

As online collaboration saves time and resources, particularly when people would otherwise have to travel long distances to participate in person, it should continue regardless of the COVID-19 pandemic. However, steps should be taken to minimize the disadvantages.

One important learning was, to start by providing a good introduction to the digital tools used in the process and continued access to digital support for those who need it.

Ideally people participating in online co-creation processes should first meet in person, to introduce themselves and make a deeper connection than they would if meeting for the first time online. This could improve understanding and willingness to listen to and consider different perspectives, which fosters collective intelligence.

A hybrid format, which involves some people participating in person and others virtually could be used for subject experts attending as guests to provide insights and information to the focus group. The information sharing and communication flows from external subject experts to the focus group members can be done online while the informal communication channels are less important. By contrast, co-creation processes which benefit from shared contributions from multiple individuals, would likely be unsuited to the hybrid format. In fact, a hybrid format may be counterproductive as it creates a sense of distance between people participating in person and those participating virtually and a different level of understanding and commitment between the two sub-groups. The session duration for online collaboration processes should take into account screen-induced fatigue. A one-day co-creation workshop is very intensive and would be very challenging online. Shorter sessions of one to two hours would be easier to manage and still allow to dive deep into the topic.

## Conclusion

Innovation is necessary to tackle the challenge of sustainability, of digitalization and of a rapidly evolving society. Geneva Macro Labs has created and designed a new model for creating responsible ventures that promote sustainability. A systemic approach was used as an enabler for collective intelligence as the driving force for innovation during the process and also as the basis for reviewing the success of the implementation. Due to the pandemic the project designed as an innovation enabler needed to rapidly innovate itself. Agile methods proved to be helpful in both, the planned innovation process as well as in adapting the process to a sudden change of the environment caused by the COVID-19 pandemic.

The authors propose a methodology that starts with a systems thinking perspective. It combines design thinking and agile development methods for harnessing collective intelligence. This creates a space for a set of experts to generate ideas and innovate beyond the mere addition of expertise. Well-orchestrated collective intelligence is a prerequisite for innovation that supports sustainable development. Systems theory, as a basis of the applied methods and attitudes of collaboration and co-creation, provides a useful framework, even if conflicts and conflicting interests arise. Systems thinking helps to consider the bigger picture, make sense of complexity through patterns and identify what is really important, in order to create a “difference that makes a difference”. The practical application of this approach in the case study showed important learnings for further optimization. The authors argue that their approach is key to fostering a successful cross-sector collaboration and enable sustainable innovation.
